# Impact of the Introduction of e-learning prior to a basic transthoracic echo course

**DOI:** 10.1186/cc14213

**Published:** 2015-03-16

**Authors:** P Madhivathanan, S Jain, D Walker

**Affiliations:** 1 Barts Health NHS Trust, London, UK; 2 Homerton University Hospitals NHS Foundation Trust, London, UK; 3 University College London Hospitals NHS Foundation Trust, London, UK

## Introduction

Focused Intensive Care Echo accreditation is a nationally approved pathway for training and accreditation in basic transthoracic echocardiography (TTE) in the UK. Recently, an e-learning module, the Intensive Care Echo and Basic Lung Ultrasound (ICE-BLU), has been introduced to facilitate TTE learning [1]. Previous work from our group has shown that incorporating simulation-based teaching elements into a basic TTE course improves candidates' satisfaction [2]. We assessed the impact of introducing the ICE-BLU e-learning programme prior to our simulation-based basic TTE course.

## Methods

**Figure 1:**
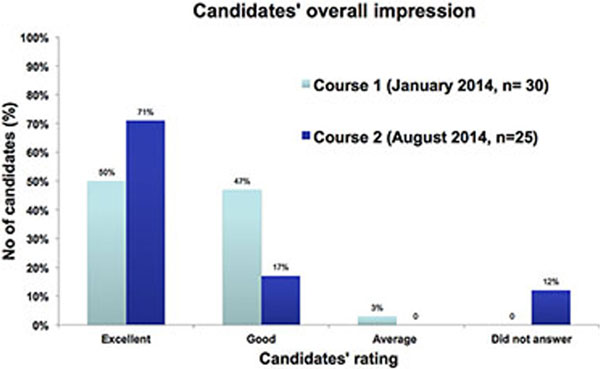


Prior to the August 2014 course, all candidates were required to complete the ICE-BLU e-learning module. On the morning of the course, the candidates completed a questionnaire to assess the impact of the e-learning module. The survey included questions on the quality of content, user friendliness, whether the content was pitched at the right level and any problems faced whilst accessing the e-learning module. We also analysed candidates' feedback from our January and August 2014 courses (Figure 1).

## Results

The response rate of the survey was 100%. Eighty per cent of candidates completed the e-learning module. The e-learning module was rated high by most candidates (80%). However, nearly one-half of the candidates faced problems accessing the module, online. Analysis of candidates' feedback (from the January and August 2014 courses) revealed that candidates' overall impression was better with the Introduction of e-learning prior to the course.

## Conclusion

Our survey has shown that the e-learning initiative was welcome by the candidates. We conclude that Introduction of e-learning prior to a simulation-based basic TTE course enhances candidates' satisfaction and feedback.
